# The impact of COVID-19 on patient engagement with primary healthcare: lessons from the saudi primary care setting

**DOI:** 10.1186/s12875-023-02131-4

**Published:** 2023-09-06

**Authors:** Alaa Alghamdi, Angus I. G. Ramsay, Ruth Abrams, Julia V. Bailey

**Affiliations:** 1 Department of Primary Care and Population Health, Institute of Epidemiology and Health Care, University College London, London, UK; 2 Department of Family and Community Medicine, King Fahad University Hospital, Imam Abdulrahman Bin Faisal University, Dammam, Saudi Arabia; 3 Department of Applied Health Research, Institute of Epidemiology and Health Care, University College London, London, UK; 4 Faculty of Health and Medical Sciences, School of Health Sciences, University of Surrey, Surrey, UK

**Keywords:** patient experience, primary healthcare, COVID-19 pandemic

## Abstract

**Background:**

There have been significant achievements in controlling COVID-19 in Saudi Arabia (SA), but as in most healthcare settings worldwide, health services have been seriously disrupted. Also, with pandemic control measures such as lockdowns and curfews, and innovations such as digital health services, the delivery of primary healthcare (PHC) services has dramatically changed. However, little is known about patients’ experiences of PHCs during the pandemic, their views on the pandemic-related interventions in SA, and patient views on impact on their medical care.

**Methods:**

Qualitative semi-structured online interviews were conducted for twenty-four Saudi patients across SA aged 18 and above who were diverse in terms of age, gender, education and health status. Data were analysed using thematic analysis yielding four major themes as an impact of COVID-19 on patient engagement with PHCs.

**Results:**

The COVID-19 pandemic has had profound mixed impacts on patient engagement with PHC in SA. Fear of infection or of breaking lockdown rules has negatively impacted the utilisation of PHCs but positively changed patients’ attitudes towards seeking medical help for self-limiting conditions. The pandemic has also negatively impacted routine preventive care at PHCs, especially for patients with long-term health conditions. The mandatory use of some digital health services in SA that existed pre-pandemic has provided patients with a perception of better care during the pandemic. Yet, a lack of awareness of optional digital health services, such as virtual clinics, hindered optimal use. Despite pandemic-related disruption of patient engagement with PHCs, the reduced waiting time in PHC centres and healthcare providers’ communication and empathy during the pandemic contributed to patients’ perceptions of better care compared to pre-pandemic. However, patients living outside the main cities in SA perceived care quality as less good during the pandemic compared to PHCs in the main cities in SA.

**Conclusion:**

The lessons learned from patients’ experiences and views of PHCs during the pandemic were beneficial in promoting patient engagement with PHCs. The digital health services mandated in response to the pandemic have accelerated digital health innovation in SA and allowed patients to recognise the benefits of digital health. This has huge potential for increasing continuous patient engagement with PHCs. Yet, patients’ experiences of digital health services during the pandemic are essential for informing appropriate implementation and utilisation of e-health services. Patients’ positive experiences of PHCs during the pandemic, such as the reduction in waiting times and the perception of improved healthcare providers’ professionalism, communication and empathy, can be built on to sustain engagement with PHC services. These findings might have significance for clinicians and policymakers to support patient engagement with PHCs, particularly in healthcare systems like SA that struggle with the overuse of emergency departments (EDs) for PHC-treatable conditions.

**Supplementary Information:**

The online version contains supplementary material available at 10.1186/s12875-023-02131-4.

## Introduction

Saudi Arabia (SA) responded to the COVID-19 crisis rapidly, initiating the highest-level public health emergency response. A range of measures were implemented, such as travel restrictions, lockdowns and curfews, social distancing, suspension of work at workplaces and roll out of COVID-19 testing [1]. The Saudi Ministry of Health (MOH) diverted resources to healthcare facilities to manage the high number of COVID-19 cases [2]. The MOH also promoted the use of many digital health applications and mobile services during the pandemic and mandated some applications, such as the tracking app Tawakkalna (Appendix 1) [3]. These initiatives helped attain a relatively low fatality rate of 1.3% compared with the international case fatality rate of 3.2% [4]. SA’s response to the pandemic places it at the highest ranks on international indexes [5] and second on the NIKKEI COVID-19 Recovery Index [6].

Despite significant achievements in controlling the COVID-19 pandemic in SA, the pandemic response measures dramatically changed the delivery of primary healthcare (PHC) services, and health services have been partially or completely disrupted, as in most health systems worldwide [7]. Pre-pandemic engagement with PHCs in SA was already poor [8], as many patients sought care at emergency departments (EDs) for conditions that would have been treatable in PHCs [9]. During the pandemic, elective visits to health services were deferred whenever possible to decrease the risk of transmitting the virus, and in-person consultations were converted to online or phone consultations, [10] forcing patients to engage with PHC services differently. Also, many patients experienced huge anxiety and distress generated by the pandemic [11] that might had an additional adverse impact on their engagement with PHCs. Few studies were conducted to measure the patients’ experiences with PHCs during the pandemic but with a quantitative design [12]. The current study, however, is planned to fill this gap and investigate more in-depth first-hand information and experience of patients.

This study aims to explore patients’ experiences and views of Saudi PHCs during the COVID-19 pandemic and how the pandemic might have impacted patient engagement with PHCs, patterns of seeking healthcare, and perceived impacts on health.

## Methods

This study was approved by the University College London ethics committee (reference no. 18,981/001). This study is part of a larger qualitative study that explores the barriers and facilitators of patient engagement with Saudi PHCs to produce recommendations to support an appropriate long-term engagement with PHCs in SA. In this paper, we focus on data that has arisen inductively from our analysis, which specifically focused on patients’ experiences with PHCs during the pandemic and the impact of COVID-19 on their engagement with PHCs.

### Study design

The study applied qualitative research methods using semi-structured interviews [13].

### Setting, sampling and recruitment

The target participants were adult Saudi patients who required PHC services (having consulted PHC centres or EDs) during the COVID-19 pandemic. To maximise sample heterogeneity, we used purposive sampling to identify Saudi patients aged 18 years and above who were diverse in terms of age, gender, education and health status (details of participants’ characteristics are available in Appendix 2). Participants from a variety of geographical locations in SA were recruited. Potential participants were approached by their care teams (including GPs, ED physicians and nurses) during healthcare consultations in PHC centres or EDs. Snowball sampling was used for groups that were harder to reach, such as those in rural areas. Participants were informed of the purpose of the study and that their participation was voluntary and would not affect their medical care. They have been assured that their participation will be kept confidential, and that the data used will be anonymized. Consent was obtained prior to each interview.

Interviews were conducted by the lead author (AA), a Saudi female clinician and researcher. Data were collected using online semi-structured interviews between February 2021 and September 2021. The interviews took place remotely using Microsoft Teams because of the restrictions imposed by the COVID-19 pandemic. Online interviews provided the opportunity to recruit participants from different regions in SA and reach participants at a convenient time and place, as well as allowing a secure way of recording the interviews. Interviews were audio-recorded with the participants’ permission and ranged from 30 to 60 min. We continued to recruit participants until we reached thematic saturation [14], with no further emerging themes.

### Interview topic guide

The topic guide for semi-structured interviews was developed to explore the patients’ experiences of PHC services and the barriers and facilitators to engagement with PHC during the COVID-19 pandemic. For ED participants who had not used PHC services, their reasons for non-attendance were explored.

### Data analysis

Audio recordings and verbatim transcripts of the interviews were used for thematic data analysis [14]. The transcripts’ accuracy was ensured by checking against audio recordings. All interviews were conducted in Arabic, and a professional transcriber and translator transcribed and translated the data into English. Three interviews were translated back into Arabic to ensure translation accuracy. The interview data were analysed iteratively to determine themes for exploration in subsequent interviews.

Audio recordings were reviewed to take notes on the participants’ tone of voice and verbal cues (by AA). The lead author, AA, immersed herself in the data by reading and rereading the transcripts and listening to the audio recordings. The codes were grouped into categories to develop an analytic framework, and preliminary themes were produced. The themes were continuously reviewed to ensure that they were coherent and that they represented the data accurately. The transcripts were coded with Quirkos software using the process described by Braun and Clark [14]. JB and AR separately undertook manual coding of one transcript and codes from this transcript were reviewed, discussed and revised, to refine the analytical coding frame, until agreement was reached. The research team discussed and refined the emergent themes.

## Results

24 patients, (15 female and 9 male), aged 18–75, participated in the study. The majority of the participants were college educated, nine were high school educated, and one was illiterate. Eight participants had long-term health conditions.

Four main themes were identified: [1] fear of visiting PHC centres during the pandemic; [2] impact on preventative care; [3] the role of online health services in patient engagement with PHC; and [4] patients’ perceptions of better care.

### Fear of visiting PHCs during the COVID-19 pandemic

While the Saudi MOH maintained health service delivery during the pandemic, many participants opted not to visit PHC centres because of their fear of exposure to COVID-19 or of breaking lockdown rules. Anxiety about visiting PHC centres persisted even after government restrictions were eased.

*‘I cannot remember when my last visit was. I have become afraid of visiting PHC units these days because of COVID-19’. (P12)*

However, the fear of infection or breaking lockdown rules appeared to change patients’ attitudes towards seeking medical help. Many participants who used to visit EDs with non-urgent -primary healthcare- conditions became more aware of when and how to seek medical help. Most of the participants preferred to self-manage their mild health concerns instead of immediately seeking PHC or EDs.

*‘Now, I deal with simple illnesses at home. I do not go to the primary clinics anymore, especially with the COVID-19 pandemic. If I have simple symptoms, such as a headache, I may visit a pharmacy to get some painkillers that can relieve my symptoms and wait until the pain subsides on its own’. (P3)*

*‘Now with the COVID-19 pandemic, if I have minor health problems, I will use natural alternatives at home like, for example, hot drinks and healthy food, unless I really need to go to the hospital, which I would do if necessary’. (P16)*

This change in patient attitude toward seeking medical help, especially regarding the visits for self-limiting conditions, was one of the positive impacts of COVID-19 identified in this research. This shift in patient medical-seeking behaviour was a possible explanation for patients’ perception of better care at PHCs during the pandemic (theme 4) and how the pandemic presented an opportunity to concentrate on the quality of care for patients requiring PHC visits.

### Impact on preventative care

The pandemic disrupted preventive screening and failed to meet healthcare needs, especially for patients with long-term health conditions.

Researcher: When was the last time you checked your blood sugar?

*Participant: I checked it last year. I was supposed to do it sooner, but the pandemic delayed my appointments for tests. They only care about dispensing medications. (P7, patient with diabetes)*

Also, annual screening for diabetes complications, such as retinopathy, diabetic nephropathy and diabetic foot, was postponed.

Children’s vaccination programmes were also affected. While most patients had no issues accessing PHCs, they experienced difficulties with the PHC system that prioritised appointment availabilities. Patients perceived less attention was given to routine vaccination appointments.

*‘They told me that the vaccinations were over and that I had to make a new appointment. This is why my children have not received their vaccinations until now, even though they will be joining school this year. My daughter still has to get one vaccination, and my son still has to get two vaccinations’. (P15)*

Besides patients’ experiences with a lack of preventive care during the pandemic, participants described some serious consequences attributed to the disruption of healthcare delivery generated by lockdown measures and prioritising COVID-19 cases.

*‘My mother has been suffering from pulmonary hyperinflation for a long time and has been following up in the PHC centre. When we planned to travel in order to finally have her lung transplantation appointment, COVID-19 broke out, and lockdowns were implemented. The surgery was postponed, and her follow-up appointments were affected too; she passed away three weeks ago’. (P15)*

The disruption of PHC delivery that mainly affected preventive care during the pandemic was one of the negative impacts of COVID-19 on patient engagement with PHC services in SA.

### Role of digital health services in patient engagement with PHC

Many participants found that the mandatory use of digital health applications during the pandemic was useful to recognise the advantages of digital health services in SA that already existed pre-pandemic. Patients highlighted the advantages of using the electronic appointment system in terms of better accessibility than the previous walk-in option or traditional appointment booking through the phone. They also found that using the online booking app reduced crowds and the long waiting time in PHC centers.

*‘The Ministry of Health has recently created marvellous apps; people are not obliged anymore to go to healthcare units and book appointments in person, as they can do this task online with just a few clicks. They can choose from nearby healthcare units. They can also select the appointment time and date. This helps save time compared with the long waiting time before. I think this is a very good improvement that I noticed during the COVID-19 pandemic’. (P11)*

In response to the pandemic restrictions, many patients had to use the medication dispensing system, i.e. electronic refill requests and medicines that can be delivered to the patient’s home or to the nearest pharmacy to minimise the risk of COVID-19 spread. Some participants felt that apps improved adherence to medications and PHC follow-ups.

*‘My father suffers from blood pressure issues. When the Wasfaty application was created, getting medications became an easier task. Even my father could get his medications on his own. With the new system, medicines are always available, and my father is taking his medications on time. I am not sure if this app was available before the pandemic, but as you know, we just realised how useful it was during the pandemic’. (P7)*

Some participants highlighted the pandemic’s advantages of imposing digital health utilisation in understanding patients’ rights, including raising complaints that support patient engagement with PHCs.

*‘It is easy to file a complaint now, especially after the emergence of the COVID-19. People have become more aware of how to file a complaint using smartphones.’ (P11)*.

The advantages of digital healthcare services during the pandemic were not limited to enabling the continuity of healthcare delivery in SA. Many patients felt that digital health services had provided them with a perception of better PHC services and eliminated some negative views about PHCs have existed pre-pandemic in SA.

*‘The situation has recently changed, especially with COVID-19. Before, we would visit private hospitals for whatever symptoms we had. But now, I feel that the situation has gotten better in healthcare units and online services. I feel that they care a lot. They are doing a good job, even if there are some shortcomings. Still, I trust the healthcare unit more now’. (P6)*

*‘Because of the COVID-19 crisis at present, they have made services available online so that we do not need to go directly to healthcare units. Every week, we can log into the available links and send messages via WhatsApp for queries or to have medications delivered at our homes. I feel that things have gotten better dramatically’. (P4)*

However, many participants’ experiences with digital health services were limited to mandatory-use apps. A profound lack of knowledge was found towards the optional digital health services.

*‘I have never used health apps. I only have Tawakkalna because it is mandatory, as you know. I have never heard about online consultations. I do not think they are available in my area’. (P22)*

As alternative options for in-person PHC delivery have shifted as much as possible to virtual clinics or teleconsultation, many participants had no experience with virtual clinics because of a lack of knowledge. Only a few participants who had a well-established rapport with their GPs benefited from virtual consultations during the pandemic.

*‘I really like my doctor; I have been following ups with her for seven years now. Because of the COVID-19 crisis, she began to communicate with me online. She is monitoring my case, asking me about my health status and whether I am stable, need to be hospitalised or need an in-person appointment. It is a good idea; I like it very much’. (P4)*

This has also shown the importance of the GP-patient therapeutic relationship to support patient engagement with PHC virtual clinics.

### Patients’ perceptions of better care

Besides the perception of better care during the pandemic due to digital health experiences, many participants felt that the quality of care during the pandemic improved in PHCs compared to pre-pandemic largely due to better organisation of PHC services. For example, many patients experienced a **reduction in the usual waiting time** in PHC centres compared to pre-pandemic. Whether the reduction was resulted from low patient utilisation of PHCs during the pandemic (as explained above) or due to MOH policy in updating appointment slots for patients as COVID-19 precautions; this has influenced the patients’ perception of better care at PHCs during the pandemic.

*‘The situation has become much better in healthcare units nowadays with COVID-19. I managed to accomplish my task quickly. I think the staff has improved considerably; maybe they made things quicker [waiting time] to let people leave as quickly as possible and avoid the spread of COVID-19’. (P11)*

Participants commented on the marked changes in PHCs administration and staffing, in particular on how PHC staff appear more professional than pre-pandemic.

*‘I know how they performed before, and it made me think that they are more professional now during the pandemic. I can see their work discipline, as all workers come at 8 AM, which did not happen before. The way medication is dispensed, the doctors’ services, the manager and his assistant… You feel that the place is well managed and supervised compared with the situation before the pandemic’. (P8)*

*“When I went before, the nurse was absent. They told me to wait, and I kept waiting for about an hour in no avail as she did not come. But this is not the case now”PT7*.

Patients found that GPs provided adequate time for examination during the pandemic, with a perception of better GPs’ communication skills and empathy. Such GPs behaviours have provided patients with a perception of better care during the pandemic.

*‘When I entered the physician’s office, she was very nice and kind. She dedicated much time to welcoming us, and she spent enough time answering some questions. My daughter was fully examined, and she kept talking to her and to me nicely, which had not been the case before. The COVID-19 crisis made the situation much better in terms of doctors’ communication, caring and empathy. She [the doctor] told me I could call a certain number so that I could know the results earlier. She was cooperative, and I liked her empathy. I was well received, and I think things changed for the better during the COVID-19 crisis’. (P11)*

*‘Usually, I do not go to healthcare units; I may go there two or three times a year just for my children’s vaccination. However, during my visits when the COVID-19 pandemic broke out, I noticed that they are more professional and caring now. Even the staff has improved their way of dealing with us’. (P14)*

Prior to the pandemic, patients believed that staff were overworked and overburdened, impeding their ability to effectively interact with and care for patients:

*“Patients want to become optimistic about their health conditions. There were few doctors who have that skill when dealing with us. I think that workload is the cause of that. Healthcare units is lack of that skill and lack of motivation. But seems better now during the pandemic”. PT9*.

*“I used to see nurses suffering from crowding and burdened by the numerous tasks that must be completed at the same time. Some of them can handle work pressure, while others cannot.”PT23*.

However, variations in PHC delivery were raised by geographically deprived patients who live out of the main cities in SA.

*‘Personally, I have experience with the healthcare units here in the south as well as in the eastern region, and I can see the huge variation between the services provided. Even now with COVID-19, I still travel for follow-ups in the eastern region while I live in the south. As I told you, with my hypothyroidism, I trust the service there’. (P22)*

Such responses demonstrated that regional PHC variances existed before the pandemic but appeared to have worsened due to the pandemic.

Due to COVID-19, many patients who resided in towns or rural areas experienced temporary site closures and relocations compared to patients in main cities who perceived better care.

*‘I used to visit a nearby healthcare unit. It was excellent, and the people there were excellent as well. But it has been dedicated to COVID-19 cases, so we have been transferred to another unit that I do not like’. (P15)*

Patients living out of the main cities also experienced limited access to COVID-19 testing points. As a result, they used PHC services for COVID-19 testing purposes, which affected other patients’ usual care at PHCs.

*‘COVID-19 has badly increased the frequency of visits to primary healthcare facilities in our area. Everyone who has even the slightest cold symptoms goes to their healthcare units because they suspect they have COVID-19. This causes overcrowding in the healthcare units. I went there three times just to get a medical swab, and the results were all negative’. (P23)*

Patients’ varying perceptions of care during the pandemic were mostly due to variations in PHC service organisation across regional areas of SA, especially in rural areas in contrast to patients’ experiences with PHCs in major cities.

## Discussion

The COVID-19 pandemic had a profound impact on patient engagement with PHC services in SA, in positive as well as negative ways.

People in SA experienced moderate to severe psychological impacts from the COVID-19 outbreak [15]. Although the COVID-19 vaccine was first introduced in Saudi Arabia in mid-December 2020 (prior to our study’s data collection) [16], our data did not reveal any effect of the COVID-19 vaccine on their fear, contrary to the findings of other studies, which found that the COVID-19 vaccine has improved people’s anxiety [17]. Our study, however, suggests that fear of exposure to COVID-19 or of breaking lockdown rules impacted on patients’ decisions to access healthcare. Fear of service engagement during the pandemic reduced healthcare utilisation by approximately a third, with greater reductions amongst people with less severe illnesses [18]. However, one beneficial effect of patients’ fear in our study was patients’ willingness to reduce unnecessary in-person visits for self-limiting conditions. The pre-pandemic engagement with PHCs in SA was poor [8] because many patients relied on EDs for primary care conditions [9]. Thus, Knowing when and how to visit PHCs or EDs and what patients can do themselves has been proven to be valuable for PHC delivery, allowing for a focus on the quality of care [19]. While data valued patient-seeking behaviour with self-limiting conditions from an economic standpoint [20], other evidence emphasises the role of community education regarding safe medication practices [21].

Therefore, many opportunities were anticipated from the changes in patients’ attitudes to seek medical help to further develop the virtual care model, especially for non-urgent health concerns and stable chronic needs. The current acceptance of and need for digital solutions have opened a window to further deploy the model instead of the traditionally preferred face-to-face contact [22]. Virtual clinics and teleconsultation during the pandemic enabled care continuity and rapidly accessible information [23]. However, the acceptability of digital solutions may present challenges because of potential conflicts with patients’ cultural, moral and religious backgrounds [24]. Culture has been identified as a greater barrier than technical matters for the utilisation of telemedicine in Middle Eastern countries [25]. Religion was among the earliest concerns of some internet users in some western communities because of the emergence of new elements like screens, cameras, and electronic audio equipment [26]. Also, patient engagement in digital solutions is dependent on access to and confidence in using technology and on perceived safety when engaging in virtual clinics [27]. However, our study found that patient disengagement in digital solutions during the pandemic such as virtual clinics was hindered by a lack of awareness rather than cultural barriers or limited access and confidence in using technology.

Since 2015, SA has made progress in implementing digital health services [28]. While the expansion of digital healthcare during the pandemic has been seen in many other countries, such as Spain, China, Canada and the UK [22] [29], the pandemic has expedited the SA’s digital transformation by **mandating** the use of many digital health apps. Findings from this study indicate that mandating digital health services allowed participants to experience many pre-existing digital health apps and that patients had positive experiences. Patient experiences of digital health services has positively influenced their views around PHC services compared to pre-pandemic. For example, patients’ experiences with the online booking system have helped reduce waiting time in PHC centres and provided a perception of better care. The implementation of health technology has helped to avoid overcrowding in health centres and to lower COVID-19 infection [22]. Our data suggest that online medication delivery has promoted patients’ adherence with their medications, and better follow-up and engagement with PHC.

Yet, findings from our study suggest that a lack of knowledge about PHC service online options impacted on engagement. Few participants were aware or had experiences with the virtual clinics. Participants who followed up with a specific healthcare provider and established a good rapport with their GPs before the pandemic were aware of online consultations and appropriately engaged with virtual clinics at PHCs. This concurs with existing evidence which suggests that continuity of care during the COVID-19 crisis was better in patients with pre-existing health conditions than patients who had no pre-existing conditions [30]. This confirms the importance of the current MOH provision for the ‘GP for each family’ scheme, which aims to provide patients with a dedicated family medicine team to support their engagement with PHCs [31]. More effort is now needed to increase patients’ knowledge about the availability of online or teleconsultation services in SA [10]. Such efforts appear to be particularly necessary for the older age group, as engagement with virtual clinics proved challenging among elderly patients in SA [32]. The COVID-19 experience stresses the importance of interactions between primary care and public health authorities to raise community awareness about innovations [19]. Social media platforms have been highly beneficial to inform people about the use of digital health services during the pandemic [24].

The COVID-19 pandemic has disrupted PHC delivery as it has stepped in to help other sectors by expanding its scope of practice and loaning staff to work in hospitals and other sectors. The hospital sector was prioritised in the supply of personal protective equipment and testing facilities where collaboration or cooperative work correction is required, rather than the loaning of personnel [19]. The pandemic has disrupted the provision of routine care, postponing many services [33]. Our study found that COVID-19 affected care for patients with non-COVID-19 conditions and impacted preventative care. Our findings are consistent with previous results that one of the most significant negative effects of the pandemic has been evident in disease screening. The disruption in PHC delivery during the pandemic significantly reduced the follow-up, screening and vaccination [34]. This decline impacted the quality of essential care for patients with chronic health problems [19].

The latest records by the WHO (April 2021)- during the COVID-19 pandemic- show that 71% of all deaths globally each year were from non-communicable diseases (NCDs), including diabetes and chronic cardiovascular and lung diseases, and more than 15 million people between the ages of 30 and 69 die from NCDs [35]. People living with NCDs were even at a higher risk of severe COVID-19-related illness and death, [36] and preventive screening remain a vital component of the response to NCDs. Continuity of preventive care at PHCs is therefore essential during pandemic times, and avoiding preventable deaths for patients is urgently needed [7].

While online health services have contributed to participants’ perceptions of better care during the pandemic, other factors have contributed as well. Our study found that patients perceived better quality of care linked to reduced waiting times in PHC centres, better staff interpersonal skills and more time for patient examination. Allocating time for patient examination has supported patients’ trust towards GPs compared to pre-pandemic. Similar to our finding, increased communication between doctors and patients has provided a perception of better trust towards doctors during the pandemic [37].

Our study focused on reporting patients’ perspectives that found better care as a result of health professionals’ empathy and care. However, healthcare providers have reported positive aspects of COVID-19, such as colleague solidarity, the establishment of well-being support structures, and feeling valued by society [38]. These positive experiences that provided a perception of better care during the pandemic should find their way into the teaching and training of health professionals and address professional needs through multidisciplinary programmes [19] to support long-term engagement with PHCs.

Our study also found a perception of unequal PHC delivery during the COVID-19 pandemic. This is attributed to geographical factors that were pre-existing but exacerbated by the pandemic, especially for disadvantaged patients living in rural areas. Our findings agree with those of a study in the US regarding the unequal distribution of COVID-19 testing points. However, in the US, many studies have highlighted the larger problem of racial/ethnic disparities in health rather than geographical disparities. COVID-19 testing centres were likelier to be in well-off suburbs of predominantly White residents than in low-income neighbourhoods that were predominantly Black [39]. Also, counties with high concentrations of non-Hispanic Blacks were disproportionately affected by COVID-19 throughout most of the pandemic [40]. In the COVID-19 pandemic, racial disparities were not a concern in our study, as SA took the initiative of offering free screening, treatment and healthcare services for all its residents, including migrant workers [41]. However, the collective response to COVID-19 must strive to demonstrate a dedication to equitable PHC services that may include any kind of health disparities—social, economic or geographical. It is essential to ensure that PHC services are provided to all those who need them and health equity must be fully integrated for routine and disaster management [42].

Overall, our findings are consistent with worldwide evidence that the pandemic has provided an opportunity to evaluate how healthcare systems and resources could be utilised more effectively [43], particularly in terms of patients’ engagement and experiences with digital health services. Post-pandemic recovery allows a re-evaluation of some PHC services. It strengthens those that provide the most value and needs, such as the need for continued preventive screening among patients. And to decrease services that give minimal or no benefit, such as unnecessary in-person appointments [33]. Thus, the pandemic has presented an opportunity for systematic reforms to quality care, [18] and to implement new strategies and care models that support the appropriateness of future visits [43]. This enables for the reduction of healthcare waste sources and improvement of healthcare quality [44].

### Strengths and Limitations

This study focused on patients’ perspectives. To our knowledge, this is the first qualitative work with SA patients to understand their experiences of PHCs during the pandemic. Future research would benefit from exploring other important domains of PHC services from other viewpoints, such as those of health professionals and policymakers.

The use of online interviews restricted research participation to individuals with Internet access and familiarity with online communication. Also, because qualitative research is not a common research method with patients in SA, it may have hampered building a rapport with patients, allowing them to speak more freely, as in face-to-face interviews. However, given the circumstances and severity of COVID-19, this was the most appropriate way to safely interview participants. Yet, the use of online interviews has enriched our data as it maximised the diversity of our sample by facilitating the recruitment of patients from various regions of SA.

The lead authors’ research positionality [14] as a young Saudi female, clinician and researcher might have influenced the recruitment process and outcome. The majority of participants involved in this study were young, educated females who ought to support the lead author, leading them to complete study. Many potential male interviewees did not proceed to the interview stage of the study as they claimed discomfort in conversing with a young woman.

The lead researcher’s previous experience as a GP meant that most participants were reluctant to share negative experiences or views toward GP services. This may place a strain on the details shared by the participants. However, this was minimised and overcome as participants were made fully aware and constantly reminded of the lead researcher’s current role as PhD researcher with no clinical commitment, even to MOH primary care services (where the study was carried out).

The strengths of this study include an in-depth collation and exploration of patients’ experiences with PHC during the COVID-19 pandemic. In addition, the lead author’s clinical background strengthens conversations with participants by adding applicable insight into the participants’ clinical experiences. Furthermore, the results of this research generate outcomes and alternative views for improving appropriate engagement with PHC centres that translate beyond the SA setting.

## Conclusion

**Fig. 1:**
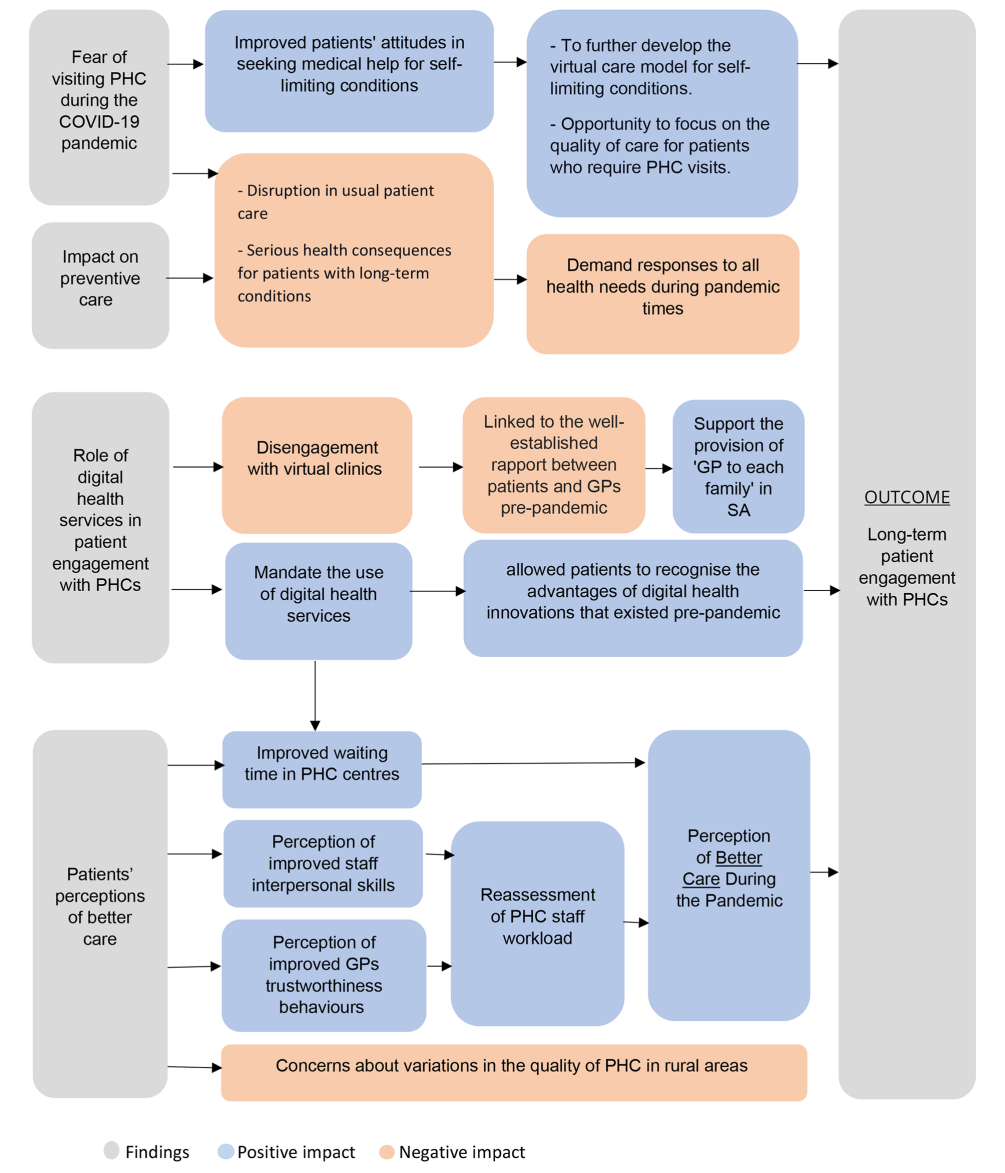
Summary of findings and lessons from patients’ experiences with PHCs in SA during the COVID-19 pandemic

There was a profound impact of COVID-19 on patient engagement with PHC services in SA in positive and negative ways (Fig. 1: Summary of findings and lessons from patients’ experiences with PHCs in SA during the COVID-19 pandemic). Patients’ experiences with PHCs during the pandemic allowed us to conclude lessons that give insight into what went well with PHC systems during the pandemic and what needs to improve to secure a better future for patient engagement with PHCs. From this study, we conclude that digital health services were useful for continuity of care during the COVID-19 pandemic. However, awareness of the different digital health services in SA hinders their optimal use, especially the virtual clinic utilisation. Engagement with virtual clinics was linked to a well-established rapport between healthcare providers and patients before the pandemic.

Preventive care is a PHC pillar that should be maintained, especially during pandemics. GPs’ efforts must be focused on adhering to screening guidelines. Health system policymakers also need to recognise that prioritising COVID-19 cases for pandemic control has other health consequences and demands responses to all health needs.

While fear impacted patient engagement with PHC, lessons could be learned from changes in patients’ attitudes toward seeking medical help for non-urgent conditions. Also, patients’ positive experiences should be considered to support patient engagement with PHC services. One explanation from our study found that perceptions of improved staff professionalism and communication have been linked to better-organised services during the pandemic, providing more timely access to quality care. Other reasons behind patients’ perceptions of better care during the pandemic need to be further explored. Yet, variations in the quality of healthcare experiences are ongoing concerns that seemed more challenging during pandemic times. Further efforts are needed to provide patients with equitable PHC experiences and eliminate health disparities.

### Importance of our research

With the comparison made with international evidence, our findings have provided evidence-based lessons on what went well during the pandemic and how to better support patient engagement with PHCs in ease and pandemic times.

### Electronic supplementary material

Below is the link to the electronic supplementary material.

Supplementary Material 1

## Data Availability

The datasets used and/or analysed during the current study are available from the corresponding author on reasonable request.
