# Supplementary material for: The impact of COVID-19 on patient engagement with primary healthcare: lessons from the saudi primary care setting
Source: BMC Prim Care. 2023 Sep 6;24:177. doi: 10.1186/s12875-023-02131-4 (PMC10483780; doi:10.1186/s12875-023-02131-4)
Supplement: Supplementary file 1 — Supplementary Material 1 [file 12875_2023_2131_MOESM1_ESM.docx]

Appendix 1– glossary of some local online health services (3)

| Tabaud | Develop​ed to track the spread of COVID-19, allowing its users to know whether they have had contact with people confirmed to be infected with COVID-19 |
| --- | --- |
| Sehhaty | Enables users to obtain health information and medical e-services, track prescribed medicine and retrieve information on and share sick leaves |
| Tawakkalna | Allows individuals to contribute to breaking the chain of infection by reporting infected cases or gatherings that violate adopted precautionary measures |
| Tetamman | Designed to provide protection and healthcare services for citizens and residents referred to domestic isolation or quarantine, as well as to maintain their safety and enhance recovery procedures |
| Sehha | Aims to provide medical consultation services electronically; allows users to have medical consultations with Ministry of Health-accredited doctors in all medical specialities |
| Mawid | Aims to enable patients and service recipients to make appointments in PHC centres in coordination with appointment staff |
| CCHI KSA | Helps users file complaints concerning health insurance |
| Tamini | Helps users obtain authorised information about registered drugs |
| Qareeboon | Provides psychological and mental counselling, which is supervised by specialised staff |

Appendix 2- Key characteristics of study participants

| Participant | Gender | Age | location | Education | Chronic health condition | Type of consultation |
| --- | --- | --- | --- | --- | --- | --- |
| Participant No.1 | Female | 35-45 | Al Baha / Makkah | Bachelor degree | No | In-person consultation |
| Participant No.2 | Female | 25-35 | Riyadh | Bachelor degree | No | In-person consultation |
| Participant No.3 | Male | 35-45 | Al Khobar | Bachelor degree | No | In-person consultation |
| Participant No.4 | Female | 62 | Dareen | Bachelor degree | Chronic health condition | Online/phone consultation |
| Participant No.5 | Female | 25-35 | Jazan / Al Khobar | High school | No | No visit during the pandemic |
| Participant No.6 | Male | 70 | Dareen | Diploma | Chronic health condition | Online/phone consultation |
| Participant No.7 | Male | 35-45 | Hail | Bachelor degree | Chronic health condition | No visit during the pandemic |
| Participant No.8 | Male | 35-45 | Hail | Bachelor degree | No | In-person consultation |
| Participant No.9 | Male | 35-45 | Hail | Bachelor degree | No | In-person consultation |
| Participant No.10 | Female | 25-35 | Qatif | Bachelor degree | No | In-person consultation |
| Participant No.11 | Female | 30 | Dammam | Bachelor degree | No | In-person consultation |
| Participant No.12 | Female | 35-45 | Dammam | Bachelor degree | No | No visit during the pandemic |
| Participant No.13 | Female | 48 | Dammam / Hajrah | Diploma | Chronic health condition | In-person consultation |
| Participant No.14 | Female | 25-35 | Dammam / Al Baha | Post-graduate degree | No | In-person consultation |
| Participant No.15 | Female | 25-35 | Jazan | High school, Diploma | No | In-person consultation |
| Participant No.16 | Female | 35-45 | Khamis Mushait | Bachelor degree | No | No visit during the pandemic |
| Participant No.17 | Female | 25-35 | Hafer Al Baten | Unschooled | No | No visit during the pandemic |
| Participant No.18 | female | 18 | Al Kharj | High school | Chronic health condition | No visit during the pandemic |
| Participant No.19 | Male | 35-45 | Mecca | High school/ diploma | Chronic health condition | In-person consultation |
| Participant No.20 | Male | 50 | Hail | High school | No | In-person consultation |
| Participant No.21 | Male | 40 | Nairyah | Post-graduate degree | No | No visit during the pandemic |
| Participant No.22 | Female | 60 | Abha | High school | Chronic health condition | No visit during the pandemic |
| Participant No.23 | Male | 70 | Khamis Mushait | High school | No | In-person consultation |
| Participant No.24 | Female | 34 | Dammam | Post-graduate degree | Chronic health condition | No visit during the pandemic |
